# The roles of environment, space, and phylogeny in determining functional dispersion of rodents (Rodentia) in the Hengduan Mountains, China

**DOI:** 10.1002/ece3.3613

**Published:** 2017-11-12

**Authors:** Yuanbao Du, Zhixin Wen, Jinlong Zhang, Xue Lv, Jilong Cheng, Deyan Ge, Lin Xia, Qisen Yang

**Affiliations:** ^1^ Key Laboratory of Zoological Systematics and Evolution Institute of Zoology Chinese Academy of Sciences Beijing China; ^2^ College of Life Science University of Chinese Academy of Sciences Beijing China; ^3^ Flora Conservation Department Kadoorie Farm and Botanic Garden Hong Kong SAR China

**Keywords:** community assembly, ecological rule, elevational gradient, functional trait

## Abstract

The recently described trait‐based approach is becoming widely popular for a mechanistic understanding of species coexistence. However, the greatest challenge in functional analyses is decomposing the contributions of different ecological and evolutionary processes (e.g., niche‐based process, neutral process, and evolutionary process) in determining trait structure. Taking rodents (Rodentia) in the Hengduan Mountains as our study model, we aim to (1) quantify the vertical patterns of functional structure for head–body length (HL), tail/body ratio (TR), animal component in diet (ACD), and all traits; (2) disentangle the relative importance of different assembly processes (environment, space, and phylogeny) in structuring trait dispersion; and (3) assess the feasibility of Bergmann's rule and Allen's rule along elevational gradient. Our results have suggested that the vertical functional structure pattern varied across these three traits, indicating distinct functional roles in the community assembly process. These nonrandom vertical patterns of HL, TR, and terminal ACD have demonstrated these traits were dominated by different ecological process along environmental gradient. In variance partitioning, high proportion of the spatial variations in trait dispersion was explained by environmental and spatial models, which have provided supporting strong evidence for niche‐based and neutral processes in leading species coexistence. Although the three traits all exhibited apparent phylogenetic signals, phylogenetic relationship within community failed to predict the spatial variations of functional dispersion, confirming the enormous inference of phylogenetic signals in predicting trait structure. By assessing the vertical patterns of HL and TR at order and family levels, we argued that functional adaptation along an environmental gradient is a surrogate of series of complex processes (e.g., environmental filtering, interspecific interaction, and neutral dispersal) acting on multiple functional axes, which results in inconsistence with the empirical rules along elevational gradient.

## 1 INTRODUCTION

Understanding the mechanism underlying community assembly is important and fundamental for proper predicting future response to ongoing climate change. Well‐known, ecological community results from series of organism–organism and organism–environment interactions, which act on multiple dimensions of functional traits. Functional traits are often considered to be measurable features of organisms that influence ecosystem‐level processes (Petchey & Gaston, 2006; Tilman, 2001). Focusing on measurable morphology, initial functional studies generalized the pattern of large‐scale morphological variation into ecological rules, offering potential to understand how organisms are restricted to the environmental conditions under which they persist (Nudds & Oswald, 2007). For example, Bergmann's rule states that mammal species tend to be larger in cooler environments (Ashton, Tracy, & Queiroz, 2000; Bergmann, 1848; Blackburn, Gaston, & Loder, 1999); Allen's rule argues that the length of appendages relative to body size is reduced in cooler environment to reduce heat loss from appendages and consequent thermoregulatory costs (Allen, 1877; Nudds & Oswald, 2007).

As the development of methodology in statistical analyses, functional studies began to relate the trait variation to the abiotic and biotic environments and space (Givnish, 1998; Jacquemyn, Micheneau, Roberts, & Pailler, 2005; Kessler, 2002). Based on different mechanistic frameworks, ecological understanding of the assembly process can be divided into two classes: the niche‐based deterministic model and the neutral model. The niche‐based deterministic model emphasizes the importance of interspecific differentiation and nonrandom responses of species to the biotic and abiotic environments (Liu, Swenson, Zhang, Ma, & Thompson, 2013). When interspecific interactions (e.g., competitive exclusion) dominate a community assembly, overdispersion should be detected in community function; if environmental filtering effects drive species coexistence, a clustered functional structure should be found in ecological communities (Swenson & Enquist, 2009). By comparison, neutral models emphasize the importance of dispersal limitation and demographic stochasticity (Geber & Griffen, 2003; Hubbell, 2001). In addition to classical ecological models, ecologists have realized that contemporary assemblages also represent a legacy of evolutionary history. Phylogenetic signals should have been imprinted on important functional characters during ongoing evolutionary processes, in terms of phylogenetic niche conservatism (PNC). Phylogenetic niche conservatism, which is defined as the tendency of species to retain ancestral ecological characteristics (Wiens & Graham, 2005), will produce a positive correlation between phylogenetic relatedness and interspecific ecological similarity. Based on this assumption, phylogenetic relatedness within a community should well predict functional diversity within an ecological community. However, some studies caution against applying phylogenetic signal and phylogenetic relatedness for predicting trait dispersion (Liu et al., 2013; Yang et al., 2014), because phylogenetic approach completely relies on phylogenetic relatedness being a strong proxy of ecological similarity (Swenson, 2013). Besides, in addition to the degree of phylogenetic conservatism, the predictive power of phylogenetic relatedness for trait dispersion also depends on the function of the trait in the assembly process (Kraft & Ackerly, 2010).

By providing a wide range of climates and habitats within a short geographical distance, the elevational gradient in a montane system is invariably favored in studies of terrestrial biodiversity (Brown, 2001; Fu et al., 2006a, 2006b; Lei, Qu, Song, Alström, & Fjeldså, 2015; McCain, 2005, 2009; Wu, Yang, et al. 2013; Wu, Colwell, et al. 2013). The Hengduan Mountains (HMs), situated at the junction between the Oriental and Palaearctic faunal realms, is a notable species‐rich and endemism‐rich biodiversity hot spot (Myers, Mittermeier, Mittermeier, da Fonseca, & Kent, 2000). In general, the high level of biodiversity in the HMs results from the contribution of its specialized geographical location, the wide range of habitats and climates along the extensive elevational gradient, the unusual topological complexity, and distinct geological events (Lei et al., 2015). Recent phylogeographical studies in the HMs have suggested that ridge‐river alternative topology may act as refugia for species during glacial periods and barriers for preventing species dispersal after glacial periods (Song et al., 2009; Fan, Liu, Liu, Zhang, & Yue, 2011; Liu et al., 2012; Lei et al., 2015; ). These unique historical events should have left distinct evolutionary imprints on the elevational patterns of trait diversity. Rodentia, the most diverse lineage of mammals (Fabre, Hautier, Dimitrov, & Douzery, 2012; Wilson & Reeder, 2005), offers an ideal model for understanding assembly processes in small mammal communities (Dreiss et al., 2015; Stevens & Gavilanez, 2015; Stevens, Gavilanez, Tello, & Ray, 2012; Stevens & Tello, 2011). Taking rodents in the HMs as our study model, we aim to (1) quantify the elevational pattern of functional dispersion in rodent communities. By applying the approach of variance partitioning, we attempt to (2) disentangle the relative importance of environment, space, and phylogeny in structuring rodent functional structure. Lastly, we calculated the mean values of two morphological traits to (3) assess the feasibility of empirical ecological rules (Bergmann's rule and Allen's rule) along the elevational gradient in the HMs.

## 2 MATERIALS AND METHODS

### 2.1 Study area and data collection

**Figure 1:**
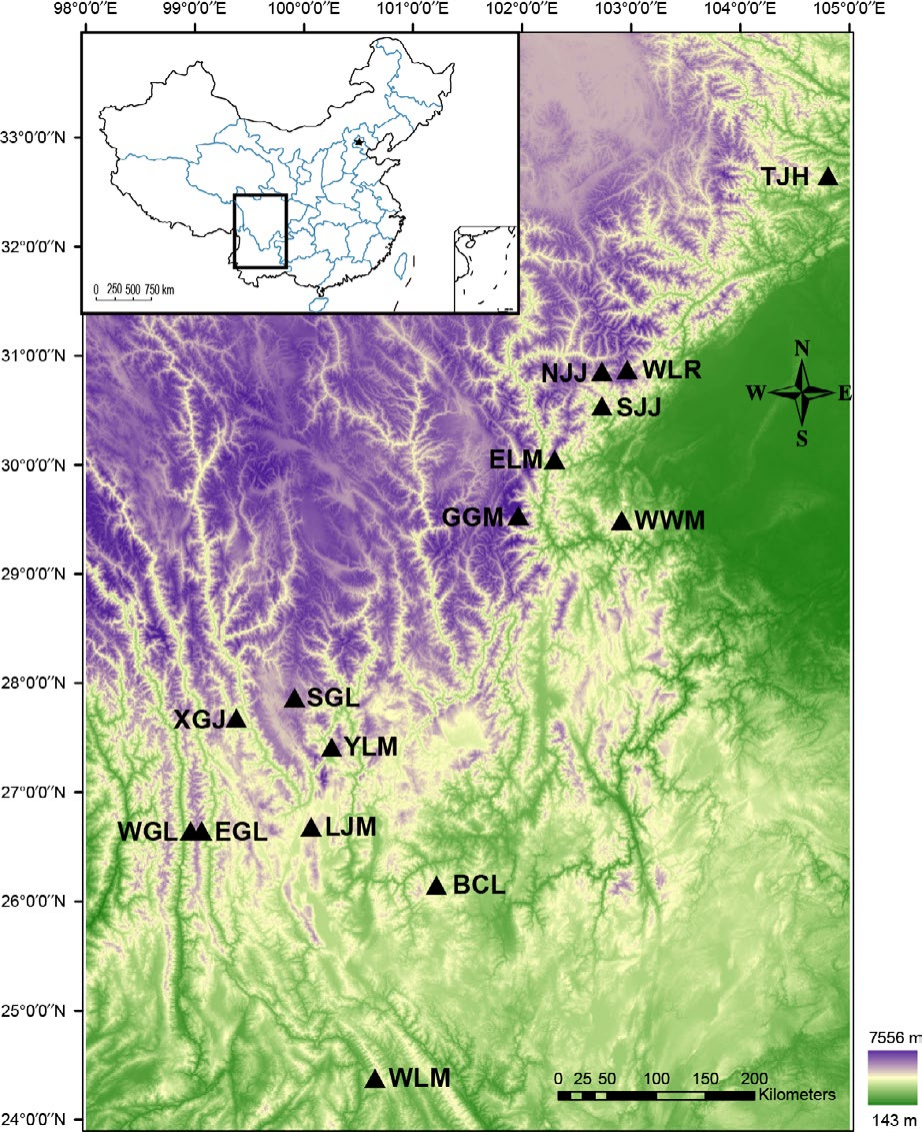
Study area and locates of 15 local field surveys in the Hengduan Mountains. The elevation is represented by gradient ramp: Green indicates lowland, while purple indicates high elevations. TJH, Tangjiahe Nature Reserve; WLR, Wolong Nature Reserve; NJJ, Northern Jiajin Mountain; SJJ, Southern Jiajin Mountain; ELM, Erlang Mountain; GGM, Gongga Mountain; WWM, Wawu Mountain; SGL, Shang‐ri La; XGJ, Xianggu Jing; YLM, Yulong Mountain; LJM, Laojun Mountain (yunnan); EGL, Eastern Gaoligong Mountain; WGL, Western Gaoligong Mountain; WLM, Wuliang Mountain; BCL, Baicaoling

From 2010 to 2015, our research team carried out four local field surveys in the HMs (22–35°N, 98–105°E; Figure 1): Mount Gongga (from May to September, 2010), Tangjiahe National Nature Reserve (from May to October, 2011), Xianggujing (from May to September, 2012; Wen et al., 2014; Wu, Yang, et al. 2013), and Wolong National Nature Reserve (October 2014 and March 2015). Samplings at these four localities were all conducted along an elevational gradient and repeated twice, covering the dry and wet seasons. Detailed information for the samplings has been presented in our previous study (Wu, Yang, et al. 2013). In addition to our field surveys, we also reviewed historical studies on rodents or small mammals in the HMs and extracted available sampling data for our analyses. In total, we collected 15 elevational transects, including 73 sampling sites and 45 rodent species of five families: Muridae (24, 53.3%), Cricetidae (13, 28.9%), Sciuridae (5, 11.1%), Dipodidae (2, 4.4%), and Spalacidae (1, 2.2%; Table S1). The taxonomy in this article follows that of Wilson and Reeder (2005) except that we recognize *Niviventer ling* as being distinct from *N. confucianus* (Bonhote, 1906; Lu et al., 2015).

Because our dataset was extracted from local surveys with different sampling biases, we transformed abundance‐weighted raw data into presence–absence data. In addition, we performed range interpolation for each species along a local elevational transect, assuming that if species occur in both higher and lower sampling sites along a local elevational gradient, they should be detected in sampling sites at the middle elevation (Wu, Yang, et al. 2013). This approach is often considered valid for predicting real animal distribution along a local vertical gradient (Wu, Yang, et al. 2013). We used this interpolated presence–absence community dataset in further mathematic analyses of phylogenetic and functional structure.

### 2.2 Phylogenetic reconstruction

**Figure 2:**
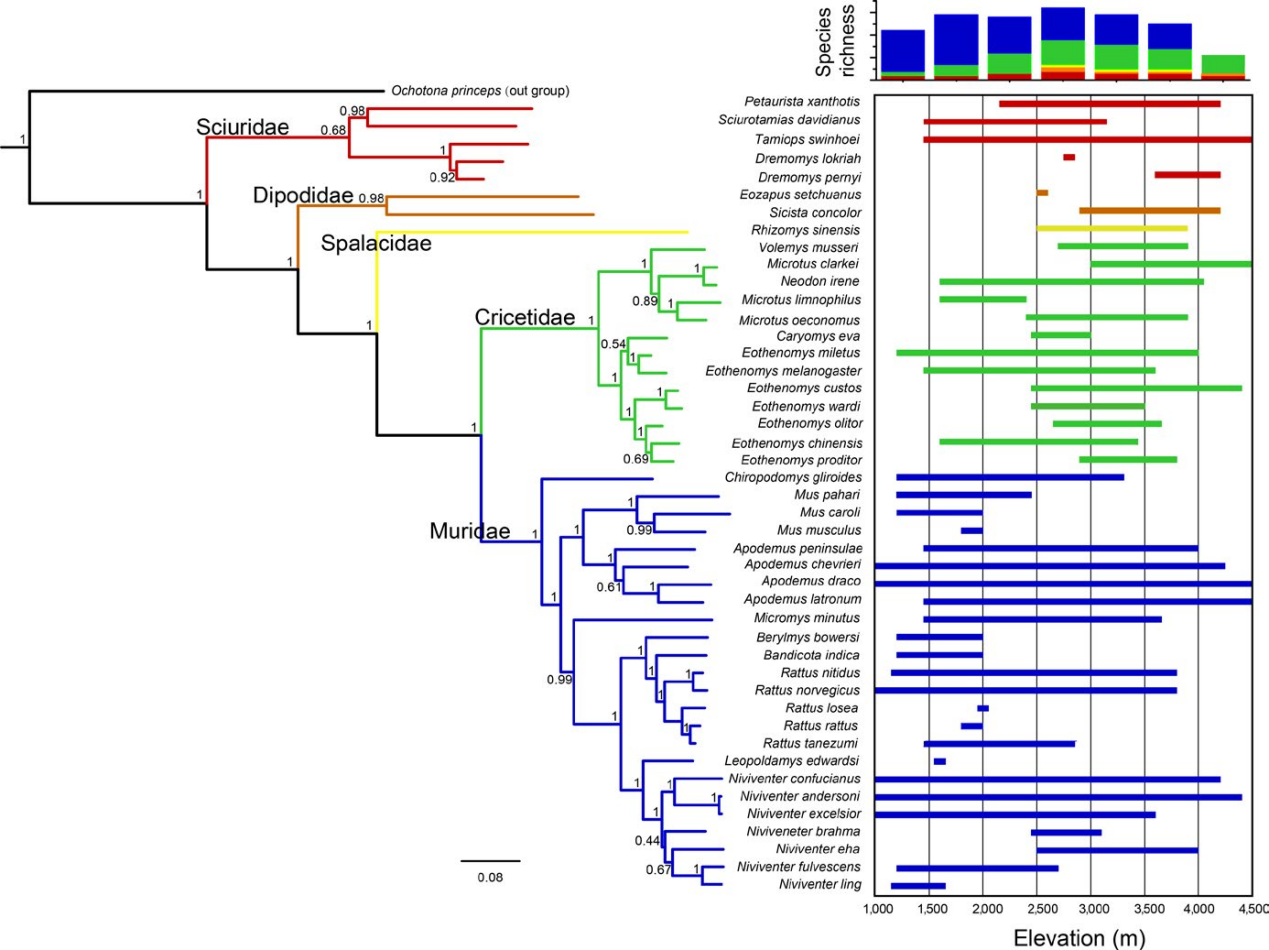
Phylogeny, elevational distribution, and species richness. The left part in this figure is the Bayesian tree reconstructed with four mitochondrial DNA genes (Cytb, CoI, 12s‐rRNA, and 16s‐rRNA) and three nuclear DNA genes (IRBP, GHR, and RAG1). The right part in this figure is the species distribution and species richness along elevational gradient

We reconstructed a phylogenetic tree using four mitochondrial DNA genes (*Cytb, CoI, 12s‐rRNA,* and *16s‐rRNA*) and three nuclear DNA genes (*IRBP, GHR,* and *RAG1*; Figure 2). All sequence data for these seven genes were obtained from GenBank (http://www.ncbi.nlm.nih.gov/genbank/; Table S2). At least one gene was available in the concatenated dataset for a species except *Vernaya fulva*. Due to the lack of sequence data in GenBank, *V. fulva* was not included in the phylogenetic tree. The sequences were aligned using the MUSCLE algorithm (Edgar, 2004) in MEGA (version 6.0; Tamura, Stecher, Peterson, Filipski, & Kumar, 2013). The best‐fit model of nucleotide substitution for each region was selected in jModelTest (version 2.1.7; Darriba, Taboada, Doallo, & Posada, 2012) under the Akaike information criterion (AIC). Phylogenetic relationships among species (concatenated *Cytb*,* CoI*,* 12s‐rRNA*,* 16s‐rRNA*,* IRBP*,* GHR,* and *RAG1*) were estimated through Bayesian inference (BI) using MrBayes (version 3.2.5; Ronquist et al., 2012). As a species related to Rodentia, *Ochotona princeps* was used as the outgroup in the phylogeny construction. Posterior distributions were calculated via the Markov chain Monte Carlo (MCMC) method with one cold chain and three heated chains for 2,000,000 generations and sampled every 1,000 generations. The first 25% of the trees were discarded as a conservative burn‐in period, and the remaining samples were used to generate a 50% majority rule consensus tree.

### 2.3 Functional traits and phylogenetic signals

As easily obtained measurements of functional characters, morphological and behavioral traits are widely used in functional analysis of animal communities (Cisneros et al., 2014; Dreiss et al., 2015; Stevens & Gavilanez, 2015). For morphological dimensions of the rodent community structure, we chose the head–body length (HL) and tail/body ratio (TR) as ecologically relevant traits, which in mammalian organisms are known to determine thermoregulation, interspecific exclusion, and resource use capacities (Bowers & Brown, 1982; Ge et al., 2014; Hayssen, 2008; Hume, Morgan, & Kenagy, 1993; Kotler, Brown, Smith, & Wirtz, 1988; Persson, 1985; see Table S3). These two morphological traits were represented by the mean values of at least eight adult specimens (four males and four females) deposited in the Institution of Zoology, Chinese Academy of Sciences (IOZCAS). For the species with no available specimens, the morphological traits were represented by data derived from historical records (Smith et al., 2010). In addition to morphological traits, foraging characters can directly reflect niche positions in feeding axes. Considering that almost all rodent species in this study are herbivorous (except for *Rattus norvegicus*), the foraging character was represented by the animal component in the diet (ACD). If a species is carnivorous, the foraging attribute is assigned “1”; otherwise, it is “0.” Foraging information was extracted from a world mammalian diet dataset (Kissling et al., 2014). By providing evidence of variations in trait evolution, phylogenetic signal detection is a widely accepted method for doing so (Losos, 2008; Revell, Harmon, & Collar, 2008). For continuous functional attributes (e.g., HL and TR), one of the most widely employed metrics for phylogenetic signals is the *K*‐statistic (Blomberg, Garland, & Ives, 2003). *K* > 1 indicates a stronger phylogenetic signal than expected from a Brownian motion model of trait evolution, and *K* < 1 indicates a weaker signal than expected (Blomberg et al., 2003). For binary variables (e.g., ACD), we used the *D* statistic (Fritz & Purvis, 2010) to verify whether the trait had a phylogenetic signal. If the trait has a phylogenetically random distribution across the phylogeny, the *D* statistic is equal or close to 1; if the observed trait is phylogenetically clumped, the value of *D* is close to or <0 (Fritz & Purvis, 2010). The detailed results of phylogenetic signal detection are presented in Table 1. All the calculations of the *K* and *D* statistics were implemented in R (version 3.1.3; R Core Team 2013) with the “picante” package (Kembel et al., 2010) and “caper” package (Orme et al., 2013), respectively.

**Table 1:** Phylogenetic signal detection for functional attributes. Significant (*p* < .05) and positive *K*‐values indicate phylogenetic signals for continuous attributes; significant (*p* < .05) and negative *D*‐values indicate phylogenetic signals for binary attributes

Attributes	Measurements	*K*/*D*‐values	*p*‐values
Head–body length (HL)	*K*‐statistic	0.424	.01
Tail/body ratio (TR)	*K*‐statistic	0.746	.001
Animal components of diet (ACD)	*D* statistic	−0.556	.001

### 2.4 Measurements for phylogenetic and trait structure

Admittedly, the observed diversity patterns and the conclusions of the analysis depend highly on the metrics used in the study (Pardo et al., 2017). However, with the rapid development of methodologies, serious metric proliferation and redundancy have been noted in both phylogenetic and functional studies (Cadotte et al., 2010; Laliberté & Legendre, 2010; Laureto, Cianciaruso, & Samia, 2015; Petchey & Gaston, 2002; Tucker et al., 2016). An appropriate metric is an important problem for any given question (Tucker et al., 2016). Thus, to eliminate metric redundancy, phylogenetic and functional alpha diversity was quantified using the standardized mean pairwise phylogenetic/trait distance (SES.MPD/SES.PW) and the standardized mean nearest neighbor phylogenetic/trait distance (SES.MNTD/SES.NN; Liu et al., 2013; Swenson, 2014). The calculations were accomplished using the following formula:

$$\text{SES}=\left({\text{Mean}}_{\mathrm{obs}}-{\text{Mean}}_{\mathrm{null}}\right)/{\text{sd}}_{\mathrm{null}}$$

where Mean_obs_ is the mean of observed measurements in a certain species assemblage, Mean_null_ is the mean of 999 randomly generated measures under the null model (independent swap; Gotelli & Entsminger, 2001), and sd_null_ is the standard deviations of 999 randomized measures. Positive *SES* values indicate overdispersion in phylogenetic or functional similarity, whereas negative values indicate phylogenetic or functional clustering (Webb, Ackerly, McPeek, & Donoghue, 2002).

### 2.5 Environmental variables and spatial eigenvectors

Eight environmental factors were applied in this study to examine their impacts on functional structure: annual mean temperature (AMT), temperature seasonality (TS), annual precipitation (AP), precipitation seasonality (PS), net primary productivity (NPP), normalized difference vegetation index (NDVI), actual evapotranspiration (AET), and potential evapotranspiration (PET). Due to the lack of detailed coordinate information, environmental variable for each sampling site was extracted using the local area and elevational range. First, we determined the local study area according to the coordinate information in each historical study. We then applied a digital elevation model (DEM) to extract the sampling area within the elevational range of each sampling site. The environmental variable was represented by the mean value within this area. Temperature and precipitation variables (AMT, TS, AP, and PS) were extracted from world climate layers (bio1, bio4, bio12, and bio15) at 30‐second resolution (http://www.worldclim.org/; Fick & Hijmans, 2017). Net primary production (NPP), normalized difference vegetation index (NDVI), actual evapotranspiration (AET), and potential evapotranspiration (PET) were obtained using MODIS products (MOD17A3, MOD13A3, and MOD16A3) accessed from the office Web site of LP DAAC (Land Processes Distributed Active Archive Center; https://lpdaac.usgs.gov/). A layer mosaic was accomplished in ENVI (ver. 4.7; ITT, 2009). Projection transformation and data extraction were carried out with ArcGIS (ver. 10.0; ESRI 2012).

Spatial eigenvectors were calculated based on a geographical distance matrix, as computed by adding the horizontal distance to the vertical distance. This transformed spatial distance matrix was then decomposed into principal coordinates of neighborhood matrix (PCNM) variables. This approach has commonly been used to transform a distance matrix into rectangular data suitable for constrained ordination or regression (Legendre et al., 2009; Liu et al., 2013; Zhang et al., 2013). These above procedures were accomplished using principal coordinate analysis (PCoA) with the R function “pcnm” in the “vegan” package.

### 2.6 Statistical analyses

We utilized simple linear regression models to ascertain the elevational patterns of functional structure (SES.PW and SES.NN). We then conducted a forward selection procedure to choose the best environmental and spatial predictive models for functional dispersion. These environmental and spatial factors as well as the phylogenetic structure (SES.MPD and SES.MNTD) were treated as explanatory variables in variance partitioning analyses. In order to assess the feasibility of Bergmann's rule and Allen's rule along vertical gradient, we log‐transformed the HL and calculated the mean values of the log‐HL and TR at family level and order level. Considering species of Sciuridae, Dipodidae, and Spalacidae occupy <20% of all rodent species in this study, the log‐HL and TR were not calculated in these three families. Simple linear regression models are used to determine trait patterns along elevational gradient. Simple linear regression, forward variable selection, and variance partitioning were all carried out in R statistical language (ver. 3.1.3; R Core Team 2013) with the “leaps” and “vegan” packages. The significance of pure and combined explanations in variance partitioning was calculated with the “rda” function in the “vegan” package.

## 3 RESULTS

### 3.1 Phylogenetic signals and vertical patterns of functional structure

**Figure 3:**
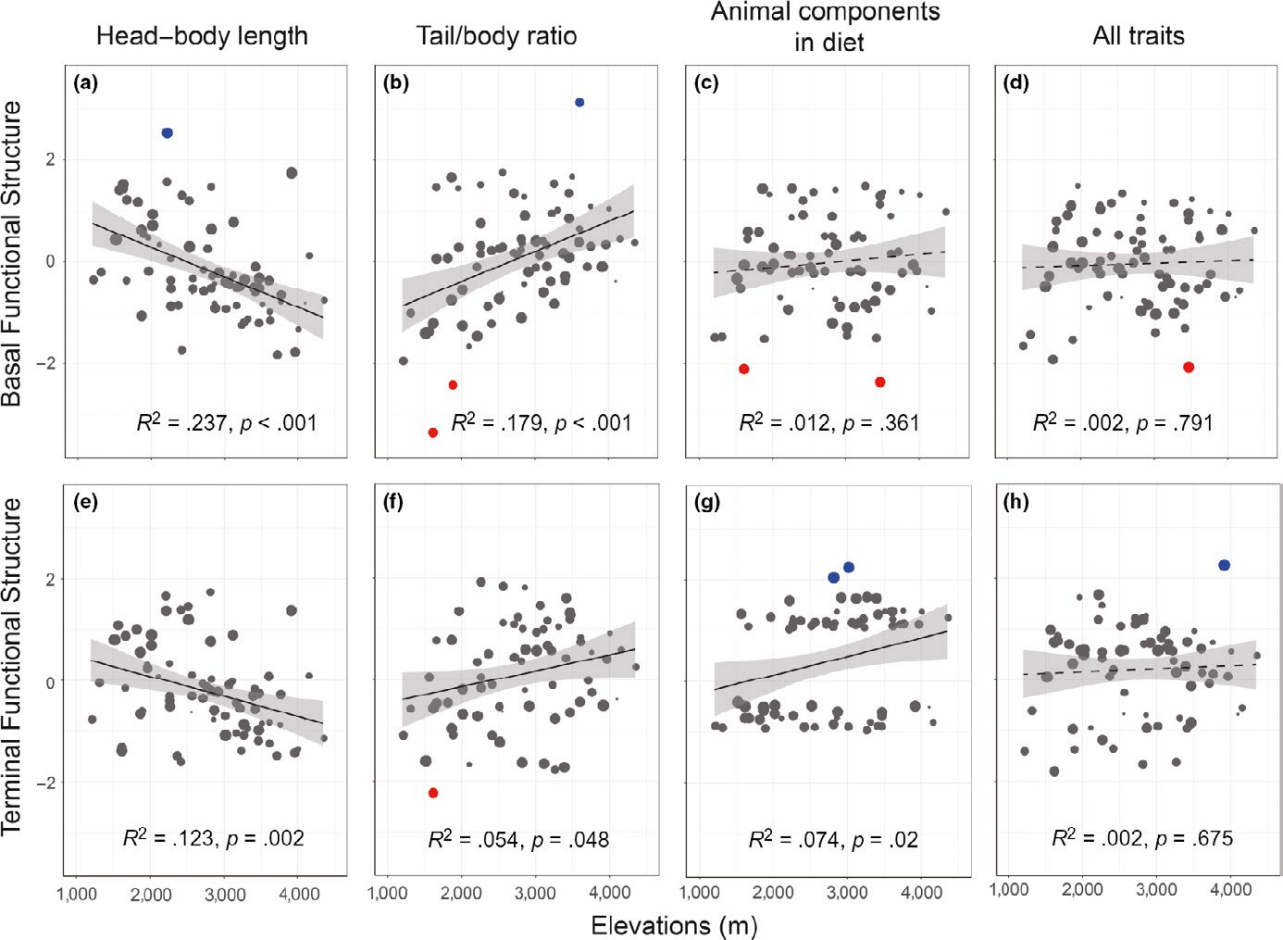
Results of simple linear regression for basal and terminal functional dispersion (SES.PW and SES.NN) of head–body length (a, e), tail/body ratio (b, f), animal components in diet (c, g), and all traits (d, h). Blue spots indicate functional overdispersion, red spots indicate functional clustering, and black spots indicate random dispersion. Spot size refers to species richness within each sampling site

The results of phylogenetic signal detection indicated that three functional attributes all exhibited a significant signal (*p* < .05; Table 1). The *K*‐values for two morphological traits were both lower than one, inferring weak signals, and the *D*‐values for ACD were lower than zero, indicating highly conservative with regard to phylogeny. The results of generalized linear regression models for trait dispersion suggested that the functional similarity of HL and TR both exhibited a nonrandom pattern along the vertical gradient (*p *<* *.05), whereas no apparent elevational pattern (*p* > .05) was detected for the functional dispersion of ACD and all traits (Figure 3).

### 3.2 Predictive models and variation partitioning

**Figure 4:**
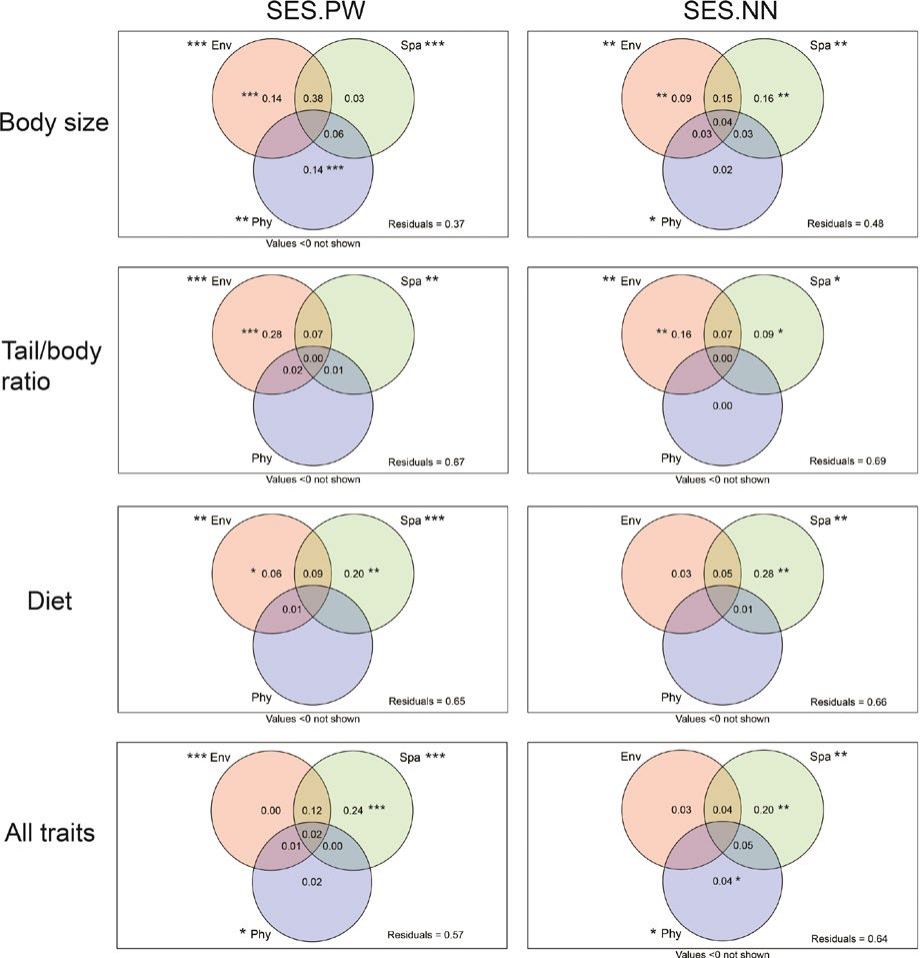
Results of variance partitioning for basal (SES.PW) and terminal functional dispersion (SES.NN). Abbreviations: Env, environmental models; Spa, spatial models; Phy, phylogenetic dispersion. Numbers within each part indicate pure or combined explanation for functional dispersion. Values <0 are not shown in these figures. Significance is signed as *p < .05; **p < .01; ***p < .001

Environmental and spatial models significantly (*p* < .05) explained the spatial variations of the basal and terminal structure of HL, TR, ACD, and all traits. Although phylogenetic models could only explain the dispersion of HL and all traits (*p* < .05), this was not the case for TR and ACD (*p* > .05). Variance partitioning analyses revealed that the pure explanation of the environment was significant (*p* < .05) for the variation of functional similarity, with the exception of ACD (terminal structure) and all traits (basal and terminal structure). In addition, the pure explanation of space was significant for almost all observed functional dispersion, except for the basal structure of HL and TR. By comparison, pure phylogenetic dispersion could only interpret the basal structure of HL and the terminal structure of all traits. The results of variance partitioning are presented in Figure 4.

### 3.3 Altitudinal patterns of body size and body shape

**Figure 5:**
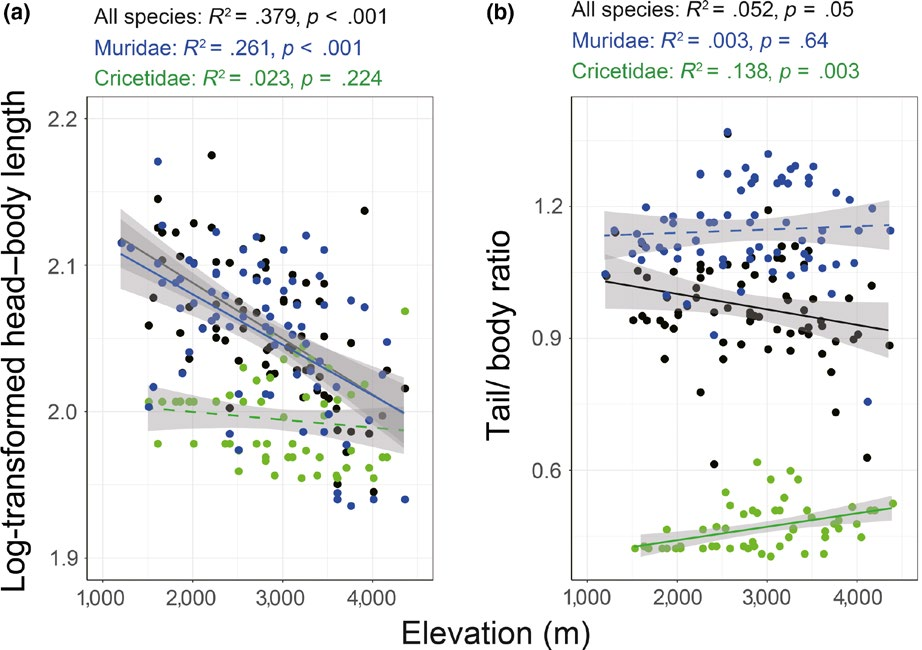
Results of simple linear regression for the means of log‐transformed head–body length and observed tail/body ratio. Spots and fitting lines in black are for all rodents. Blue and green indicate that for Muridae and Cricetidae, respectively

The means of log‐transformed head–body length (HL) at order level decreased with increasing elevation (*R* ^2^ = 0.379, *p* < .001), which was inconsistent with the expectation of Bergmann's rule (Figure 5a). Conversely, the means of the tail/body ratio (TR) at order level exhibiting a decreasing pattern along the altitudinal gradient (*R* ^2^ = 0.052, *p* = .05; Figure 5b), which was weakly consistent with Allen's rule. At the family level, log‐HL of murine species significantly decreased with increasing elevation (*R* ^2^ = 0.261, *p* < .001), but no apparent pattern could be detected in Cricetidae (*R* ^2^ = 0.023, *p* = .224; Figure 5a); the pattern of TR in Muridae was not significant with regard to elevational gradient (*R* ^2^ = 0.003, *p* = .64), while TR in Cricetidae significantly increased as increasing elevation (*R* ^2^ = 0.138, *p* = .003; Figure 5b).

## 4 DISCUSSION

### 4.1 Elevational patterns of functional structure

In this study, we quantified the functional structure along an elevational gradient for each and all traits. Distinct vertical patterns of trait diversity suggested diverse functional roles displayed by functional attributes in the community assembly process. This is because the relative importance of structuring mechanisms (e.g., environmental filtering, interspecific competition, and neutral dispersal) may vary between functional components; thus, decomposing the functional dimension into constituent components may identify opposing effects of ecological processes on functional dispersion (Cisneros et al., 2014). For instance, the overdispersed HL and clustered TR at low elevations demonstrated that an opposite effect (e.g., competitive exclusion and environmental filtering) acted on these two traits, which is the reason why no obvious elevational pattern was detected in the dispersion of all traits (d and h in Figure 3). In contrast, the vertical patterns of functional dispersion indicated that the functional role of one trait also varied along the environmental gradient. Relating the vertical pattern of functional dispersion to the empirical framework proposed by Kraft and Ackerly (2010), the dispersion of HL at low elevations and TR/ACD on high mountaintops should be strongly affected by interspecific exclusion or density constraint, producing an overdispersed status. Nonetheless, environmental filtering may be more important for HL at high elevations and TR/ACD in lowlands, producing a clustered dispersion.

### 4.2 Variance partitioning for functional structure

The central aim of this study was to disentangle the relative importance of environment, space, and phylogeny in structuring the spatial variation of rodent functional dispersion. Previous empirical studies have argued that the abiotic environment plays an important role in structuring community assembly (Kraft, Valencia, & Ackerly, 2008; Swenson & Enquist, 2007, 2009), although this inference may only apply when considering the fact of strong spatial autocorrelation in the abiotic environment (Liu et al., 2013). Coupled with the development of functional analyses, there has been a rapidly increasing interest in phylogenetic analyses for revealing community assembly processes (Cardillo, 2011; Cardillo, Gittleman, & Purvis, 2008; Stevens et al., 2012; Swenson, 2011; Swenson, Enquist, Pither, Thompson, & Zimmerman, 2006; Swenson, Enquist, Thompson, & Zimmerman, 2007; Webb, 2000; Webb et al., 2002). Based on the hypotheses of phylogenetic niche conservatism, these phylogenetic analyses assumed that the functional similarity of important traits in the community assembly process has a strong correlation with phylogenetic distance. If this assumption is met, the spatial variation in trait dispersion should be well predicted by the spatial variation in phylogenetic dispersion (Liu et al., 2013).

The three functional traits (HL, TR, and ACD) examined in this work all showed significant signals with regard to phylogeny. Exceeding our expectation, the pure contribution of phylogenetic dispersion was not significant for explaining the spatial variations of trait dispersion (except for the basal structure of HL and the terminal structure of all traits). The contradiction between the results of phylogenetic signal detection and explanatory models can likely be attributed to erroneous inferences of phylogenetic signals on trait dispersion (Swenson & Enquist, 2009). By comparing phylogenetic and trait dispersion, previous studies have argued that functional dispersion of a phylogenetically conserved trait may be inconsistent with regard to phylogenetic dispersion due to significant but weak signals in functional attributes (Swenson & Enquist, 2009; Yang et al., 2014). Our results confirmed this inference, indicating that more attention should be paid to applying phylogenetic signals to infer functional dispersion (Liu et al., 2013; Swenson & Enquist, 2009).

Among three dimensions of predictive models, pure and combined spatial models explained the highest proportion of the spatial variations in trait dispersion, suggesting the important contributions of a neutral process in community construction. Following with spatial models, environmental models were also significant for explaining trait diversity (except for the terminal structure of diet and all traits), indicating a secondary importance of nonrandom processes in community construction. Besides, it is worth noting that the combination of spatial and environmental models contributed to a fairly large proportion of the explanation, demonstrating significant spatial autocorrelation in environmental variables. An alternative interpretation for this result is that this work failed to quantify the effect of interspecific exclusion, which will reduce the magnitude of environmental filtering and enhance the influence of neutral processes.

### 4.3 Bergmann's rule and Allen's rule along the elevational gradient

These two empirical hypotheses predict that body size should increase and the tail/body ratio should decrease from low to high elevations. According to the results in this study, we found that the body size of rodents, as represented by the mean log‐transformed HL, decreased with increasing elevation, which is inconsistent with Bergmann's rule at the community level. The decreasing pattern of TR of rodents weakly met the expectation of Allen's rule. But the TR at family level could not support Allen's rule (Figure 5b). These results indicate that ecological adaptation (e.g., thermoregulation) to the local environment is a complex response occurring at multiple functional axes (Kotler et al., 1988). During long evolutionary processes, niche partitioning among lineages produces distinct physiological or ecological constraints (Brehm & Fiedler, 2004). Among 45 rodent species in this study, more than 80% (38 species) are species of Cricetidae and Muridae. Cricetidae species with a smaller body size and a lower tail/body ratio mainly colonize middle and high elevations, whereas murine species with a larger body size and a higher tail/body ratio occupy the entire elevational gradient, with a species richness peak at mid‐low elevations (Figures 2 and 5). The decreasing HL along the altitudinal gradient maybe does not indicate a “true” pattern but rather a side effect of niche partitioning between Cricetidae and Muridae (Brehm & Fiedler, 2004). Indeed, there is no evidence that Cricetidae species are successful on high cold‐wet mountaintops due to their small body sizes, and at high elevations, members of *Eothenomys* (Cricetidae) tend to maintain a larger body size than that at lower elevations (Mu & Zhu, 2015;). Back to the definitions of these two ecological rules, they both reflect morphological response to the filtering effect from high to low temperature. However, apparent elevational patterns of functional dispersion in HL and TR have suggested distinct assembly processes (e.g., environmental filtering, interspecific interaction and neutral dispersal) leading trait composition in different environments. In other words, environmental filtering is not always the dominate driver in structuring trait dispersion. This may be another reason that rodent morphology along altitudinal gradient does not support for Bergmann's rule and Allen's rule along altitudinal gradient.

## 5 CONCLUSION

This work presents an empirical study of rodent functional dispersion in a subtropical montane system. As a result of distinct functional responses to multiple ecological and evolutionary processes, vertical patterns of trait dispersion varied across three functional attributes, indicating distinct roles of functional traits in rodent assembly processes. Following with previous functional studies, we decomposed the spatial variations of trait dispersion into environment, space, and phylogeny. Being consistent with previous inference, community trait diversity was highly related to pure and combined environmental and spatial variations. However, phylogenetic relationship within the community was fairly weak for predicting trait diversity, which highlighted the erroneous inference of phylogenetic signals in trait diversity inference. In addition, our results emphasized the systematic influence of a lack of an appropriate quantification for interspecific exclusion, reducing the explanatory strength of the environment and exaggerating the effect of neutral processes. Lastly, by assessing Bergmann's rule and Allen's rule along the elevational gradient, we argue that ecological adaptation to the local environment is a complex process acting on multiple facets of functional characters. The findings presented here provide fresh empirical evidence for a mechanistic understanding of animal community construction, especially in mountainous regions.

## CONFLICT OF INTEREST

None declared.

## AUTHOR CONTRIBUTION

Y.B.D., Z.X.W., D.Y.G., L.X., and Q.S.Y. conceived of the ideas and designed the study; Y.B.D. and Z.X.W. contributed to the field survey and data collection; Y.B.D., J.L.Z., X. L, and J.L.C. analyzed data; Y.B.D. wrote the manuscript with the help of all authors.

## DATA ACCESSIBILITY

All data used in this manuscript are present in the supporting information.
