# Supplementary material for: The roles of environment, space, and phylogeny in determining functional dispersion of rodents (Rodentia) in the Hengduan Mountains, China
Source: Ecol Evol. 2017 Nov 12;7(24):10941–51. doi: 10.1002/ece3.3613 (PMC5743695; doi:10.1002/ece3.3613)
Supplement: Supplementary file 1 [file ECE3-7-10941-s001.docx]

**Supporting information:**

**Table S1.** Detailed information for 74 sampling sites: middle elevations, elevational range, longitudes, latitudes and species presence/absence. Species presence is signed with ‘√’; species absence is marked with ‘-’.

|  | Tangjiahe Nature reserve (TJH) | | | | Wolong Nature reserve (WLR) | | | | | | | |
| --- | --- | --- | --- | --- | --- | --- | --- | --- | --- | --- | --- | --- |
| Middle elevation (m) | 1500 | 1850 | 2200 | 2550 | 1550 | 1650 | 1800 | 2000 | 2200 | 2500 | 2800 | 3500 |
| Elevational range (m) | 100 | 100 | 100 | 100 | 100 | 100 | 100 | 100 | 100 | 100 | 100 | 100 |
| Latitude (N) (degree) | 32.6 | 32.63 | 32.64 | 32.65 | 31.08 | 31.12 | 31.09 | 31.03 | 30.99 | 30.99 | 30.86 | 30.88 |
| Longitude (E) (degree) | 104.83 | 104.84 | 104.82 | 104.8 | 103.32 | 103.23 | 103.24 | 103.18 | 103.14 | 103.16 | 102.97 | 102.96 |
| Reference | *f.* | | | | *Field survey* | | | | | | | |
| *Chiropodomys gliroides* | - | - | - | - | - | - | - | - | - | - | - | - |
| *Apodemus chevrieri* | √ | √ | √ | - | √ | √ | √ | √ | √ | √ | √ | - |
| *Apodemus peninsulae* | √ | √ | - | - | - | - | - | - | - | - | - | - |
| *Apodemus draco* | √ | √ | √ | - | √ | √ | √ | √ | √ | √ | √ | - |
| *Apodemus latronum* | √ | √ | √ | √ | √ | √ | √ | √ | √ | √ | √ | √ |
| *Mus caroli* | - | - | - | - | - | - | - | - | - | - | - | - |
| *Mus musculus* | - | - | - | - | - | - | - | - | - | - | - | - |
| *Mus pahari* | - | - | - | - | - | - | - | - | - | - | - | - |
| *Bandicota indica* | - | - | - | - | - | - | - | - | - | - | - | - |
| *Rattus losea* | - | - | - | - | - | - | - | - | - | - | - | - |
| *Rattus rattus* | - | - | - | - | - | - | - | - | - | - | - | - |
| *Rattus tanezumi* | √ | √ | √ | - | - | - | - | √ | - | - | - | - |
| *Rattus nitidus* | - | - | - | - | - | - | - | - | - | - | - | - |
| *Rattus norvegicus* | - | - | - | - | - | √ | √ | √ | - | - | - | - |
| *Berylmys bowersi* | - | - | - | - | - | - | - | - | - | - | - | - |
| *Niviventer andersoni* | - | √ | √ | √ | √ | √ | √ | √ | √ | - | - | - |
| *Niviventer excelsior* | - | - | - | - | - | - | - | - | - | - | - | - |
| *Niviventer confucianus* | √ | √ | √ | √ | √ | √ | √ | √ | √ | √ | √ | - |
| *Niviventer eha* | - | - | - | - | - | - | - | - | - | - | - | √ |
| *Niviventer fulvescens* | - | - | - | - | - | - | - | - | - | - | - | - |
| *Niviventer ling* | √ | - | - | - | √ | - | - | - | - | - | - | - |
| *Niviveneter brahma* | - | - | - | - | - | - | - | - | - | - | - | - |
| *Leopoldamys edwardsi* | - | - | - | - | - | - | - | - | - | - | - | - |
| *Micromys minutus* | √ | - | - | - | √ | - | - | - | - | - | - | - |
| *Caryomys eva* | - | - | - | √ | - | - | - | - | - | √ | - | - |
| *Eothenomys miletus* | - | - | - | - | - | - | - | - | - | - | - | - |
| *Eothenomys melanogaster* | √ | √ | - | - | - | √ | √ | √ | - | - | - | - |
| *Eothenomys chinensis* | - | - | - | - | - | - | - | - | - | - | - | - |
| *Eothenomys custos* | - | - | - | - | - | - | - | - | - | - | - | - |
| *Eothenomyswardi* | - | - | - | - | - | - | - | - | - | - | - | - |
| *Eothenomys olitor* | - | - | - | - | - | - | - | - | - | - | - | - |
| *Eothenomys proditor* | - | - | - | - | - | - | - | - | - | - | - | - |
| *Microtus limnophilus* | - | - | - | - | - | - | - | - | - | - | - | - |
| *Microtus clarkei* | - | - | - | - | - | - | - | - | - | - | - | - |
| *Neodon irene* | - | - | - | - | - | - | - | - | - | - | - | - |
| *Microtus oeconomus* | - | - | - | - | - | - | - | - | - | - | - | √ |
| *Volemys musseri* | - | - | - | - | - | - | - | - | - | - | - | - |
| *Dremomys lokriah* | - | - | - | - | - | - | - | - | - | - | - | - |
| *Dremomys pernyi* | - | - | - | - | - | - | - | - | - | - | - | - |
| *Tamiops swinhoei* | √ | √ | √ | - | - | - | - | - | - | - | - | - |
| *Sciurotamias davidianus* | √ | √ | √ | - | √ | √ | √ | √ | √ | √ | √ | - |
| *Petaurista xanthotis* | - | - | √ | √ | - | - | - | - | - | - | - | - |
| *Rhizomys sinensis* | - | - | - | √ | - | - | - | - | - | - | - | - |
| *Eozapus setchuanus* | - | - | - | - | - | - | - | - | - | - | - | - |
| *Sicista concolor* | - | - | - | √ | - | - | - | - | - | - | - | - |

Continued

|  | Northern Jiajin Mountain (NJJ) | | | | | Southern Jiajin Mountain (SJJ) | | | | | | |
| --- | --- | --- | --- | --- | --- | --- | --- | --- | --- | --- | --- | --- |
| Middle elevation (m) | 2550 | 2850 | 3150 | 3450 | 3600 | 1650 | 1950 | 2250 | 2550 | 2850 | 3150 | 3450 |
| Elevational range (m) | 300 | 300 | 300 | 300 | 300 | 300 | 300 | 300 | 300 | 300 | 300 | 300 |
| Latitude (N) (degree) | 30.70 ~ 31.02 | | | | | 30.38 ~ 30.7 | | | | | | |
| Longitude (E) (degree) | 102.50 ~ 102.95 | | | | | 102.50 ~ 102.95 | | | | | | |
| Reference | *e.* | | | | | *e.* | | | | | | |
| *Chiropodomys gliroides* | - | - | - | - | - | - | - | - | - | - | - | - |
| *Apodemus chevrieri* | - | - | - | - | - | √ | √ | √ | √ | √ | - | - |
| *Apodemus peninsulae* | - | - | - | - | - | - | - | - | - | - | - | - |
| *Apodemus draco* | - | √ | √ | √ | √ | - | √ | √ | √ | √ | √ | √ |
| *Apodemus latronum* | - | - | √ | √ | - | - | - | √ | √ | √ | √ | √ |
| *Mus caroli* | - | - | - | - | - | - | - | - | - | - | - | - |
| *Mus musculus* | - | - | - | - | - | - | - | - | - | - | - | - |
| *Mus pahari* | - | - | - | - | - | - | - | - | - | - | - | - |
| *Bandicota indica* | - | - | - | - | - | - | - | - | - | - | - | - |
| *Rattus losea* | - | - | - | - | - | - | - | - | - | - | - | - |
| *Rattus rattus* | - | - | - | - | - | - | - | - | - | - | - | - |
| *Rattus tanezumi* | - | - | - | - | - | - | - | - | - | - | - | - |
| *Rattus nitidus* | - | - | - | - | - | - | - | - | - | - | - | - |
| *Rattus norvegicus* | - | - | - | - | - | - | - | - | - | - | - | - |
| *Berylmys bowersi* | - | - | - | - | - | - | - | - | - | - | - | - |
| *Niviventer andersoni* | - | - | - | √ | - | √ | √ | √ | √ | √ | √ | - |
| *Niviventer excelsior* | √ | √ | √ | √ | - | √ | √ | √ | √ | √ | √ | √ |
| *Niviventer confucianus* | √ | √ | √ | √ | - | √ | √ | √ | √ | √ | √ | - |
| *Niviventer eha* | - | - | - | - | - | - | - | - | - | - | - | - |
| *Niviventer fulvescens* | - | - | - | - | - | - | - | - | - | - | - | - |
| *Niviventer ling* | - | - | - | - | - | - | - | - | - | - | - | - |
| *Niviveneter brahma* | - | - | - | - | - | - | - | - | - | - | - | - |
| *Leopoldamys edwardsi* | - | - | - | - | - | - | - | - | - | - | - | - |
| *Micromys minutus* | - | - | - | - | - | - | √ | - | - | - | - | - |
| *Caryomys eva* | - | - | - | - | - | - | - | - | - | √ | - | - |
| *Eothenomys miletus* | - | - | - | - | - | - | - | - | - | - | - | - |
| *Eothenomys melanogaster* | - | - | - | - | - | √ | √ | √ | √ | √ | - | - |
| *Eothenomys chinensis* | - | - | - | - | - | - | - | - | - | - | - | - |
| *Eothenomys custos* | - | - | - | - | - | - | - | - | - | - | - | - |
| *Eothenomyswardi* | - | - | - | - | - | - | - | - | - | - | - | - |
| *Eothenomys olitor* | - | - | - | - | - | - | - | - | - | - | - | - |
| *Eothenomys proditor* | - | - | - | - | - | - | - | - | - | - | - | - |
| *Microtus limnophilus* | - | - | - | - | - | - | - | - | - | - | - | - |
| *Microtus clarkei* | - | - | - | - | - | - | - | - | - | - | - | - |
| *Neodon irene* | - | - | - | √ | √ | - | - | - | - | - | - | √ |
| *Microtus oeconomus* | - | - | - | - | √ | - | - | - | - | - | - | - |
| *Volemys musseri* | - | - | √ | √ | √ | - | - | - | - | √ | √ | √ |
| *Dremomys lokriah* | - | - | - | - | - | - | - | - | - | - | - | - |
| *Dremomys pernyi* | - | - | - | - | - | - | - | - | - | - | - | - |
| *Tamiops swinhoei* | - | - | - | - | - | - | - | - | - | - | √ | √ |
| *Sciurotamias davidianus* | - | - | - | - | - | - | - | - | - | - | - | - |
| *Petaurista xanthotis* | - | - | - | - | - | - | - | - | - | - | - | - |
| *Rhizomys sinensis* | - | - | - | - | - | - | - | - | - | - | - | - |
| *Eozapus setchuanus* | - | - | - | √ | √ | - | - | - | - | - | - | - |
| *Sicista concolor* | - | - | - | - | √ | - | - | - | - | - | - | - |

Continued

|  | Erlang Mountain (ELM) | | | | | Gongga Mountain (GGM) | | | | | | | |
| --- | --- | --- | --- | --- | --- | --- | --- | --- | --- | --- | --- | --- | --- |
| Middle elevation (m) | 1950 | 2250 | 2550 | 2850 | 3219 | 1200 | 1600 | 2000 | 2400 | 2800 | 3200 | 3600 | 4000 |
| Elevational range (m) | 300 | 300 | 300 | 300 | 437 | 100 | 100 | 100 | 100 | 100 | 100 | 100 | 100 |
| Latitude (N) (degree) | 29.90 ~ 30.20 | | | | | 29.61 | 29.64 | 29.6 | 29.59 | 29.58 | 29.57 | 29.55 | 29.54 |
| Longitude (E) (degree) | 102.20 ~ 102.40 | | | | | 102.17 | 102.13 | 102.07 | 102.04 | 102.03 | 101.99 | 101.97 | 101.96 |
| Reference | *d.* | | | | | *g. f.* | | | | | | | |
| *Chiropodomys gliroides* | - | - | - | - | - | - | - | - | - | - | - | - | - |
| *Apodemus chevrieri* | - | - | - | - | - | √ | √ | √ | - | - | - | - | - |
| *Apodemus peninsulae* | - | - | - | - | - | - | √ | √ | √ | √ | √ | - | - |
| *Apodemus draco* | √ | √ | √ | √ | √ | √ | √ | √ | √ | √ | √ | √ | √ |
| *Apodemus latronum* | - | - | - | - | √ | - | √ | √ | √ | √ | √ | √ | √ |
| *Mus caroli* | - | - | - | - | - | - | - | - | - | - | - | - | - |
| *Mus musculus* | - | - | - | - | - | - | - | - | - | - | - | - | - |
| *Mus pahari* | - | - | - | - | - | - | - | - | - | - | - | - | - |
| *Bandicota indica* | - | - | - | - | - | - | - | - | - | - | - | - | - |
| *Rattus losea* | - | - | - | - | - | - | - | √ | - | - | - | - | - |
| *Rattus rattus* | - | - | - | - | - | - | - | - | - | - | - | - | - |
| *Rattus tanezumi* | - | - | - | - | - | - | - | - | - | - | - | - | - |
| *Rattus nitidus* | - | - | - | - | - | √ | - | - | - | - | - | - | - |
| *Rattus norvegicus* | - | - | - | - | - | √ | √ | √ | - | - | - | - | - |
| *Berylmys bowersi* | - | - | - | - | - | - | - | - | - | - | - | - | - |
| *Niviventer andersoni* | √ | √ | √ | - | - | - | - | √ | √ | √ | √ | - | - |
| *Niviventer excelsior* | - | √ | √ | √ | √ | - | - | - | - | - | - | - | - |
| *Niviventer confucianus* | - | - | - | - | - | √ | √ | √ | √ | √ | √ | - | - |
| *Niviventer eha* | - | - | - | - | - | - | - | - | - | - | - | - | - |
| *Niviventer fulvescens* | - | - | - | - | - | - | - | - | - | - | - | - | - |
| *Niviventer ling* | - | - | - | - | - | √ | √ | - | - | - | - | - | - |
| *Niviveneter brahma* | - | - | - | - | - | - | - | - | - | - | - | - | - |
| *Leopoldamys edwardsi* | - | - | - | - | - | - | √ | - | - | - | - | - | - |
| *Micromys minutus* | - | - | - | - | - | - | - | - | - | - | - | - | - |
| *Caryomys eva* | - | - | - | - | - | - | - | - | - | - | - | - | - |
| *Eothenomys miletus* | - | - | - | - | - | - | √ | √ | √ | √ | - | - | - |
| *Eothenomys melanogaster* | √ | √ | √ | - | - | - | - | - | - | - | - | - | - |
| *Eothenomys chinensis* | - | - | √ | √ | √ | - | - | - | - | - | - | - | - |
| *Eothenomys custos* | - | - | - | - | - | - | - | - | - | - | - | - | - |
| *Eothenomyswardi* | - | - | - | - | √ | - | - | - | - | - | - | - | - |
| *Eothenomys olitor* | - | - | - | - | - | - | - | - | - | - | - | - | - |
| *Eothenomys proditor* | - | - | - | - | - | - | - | - | - | - | - | - | - |
| *Microtus limnophilus* | - | - | - | - | - | - | - | - | - | - | - | - | - |
| *Microtus clarkei* | - | - | - | - | - | - | - | - | - | - | - | - | - |
| *Neodon irene* | - | - | - | - | - | - | - | - | - | √ | √ | √ | √ |
| *Microtus oeconomus* | - | - | - | - | - | - | - | - | - | - | - | - | - |
| *Volemys musseri* | - | - | - | - | - | - | - | - | - | - | - | - | - |
| *Dremomys lokriah* | - | - | - | - | - | - | - | - | - | - | - | - | - |
| *Dremomys pernyi* | - | - | - | - | - | - | - | - | - | - | - | - | - |
| *Tamiops swinhoei* | - | - | - | - | - | - | - | - | - | - | √ | √ | - |
| *Sciurotamias davidianus* | - | - | - | - | - | - | √ | √ | √ | - | - | - | - |
| *Petaurista xanthotis* | - | - | - | - | - | - | - | - | - | - | - | - | - |
| *Rhizomys sinensis* | - | - | - | - | - | - | - | - | - | - | - | - | - |
| *Eozapus setchuanus* | - | - | - | - | - | - | - | - | - | - | - | - | - |
| *Sicista concolor* | - | - | - | - | - | - | - | - | - | - | - | - | - |

Continued

|  | Wawu Mountian (WWM) | | | | Shang-ri La (SGL) | | | | |
| --- | --- | --- | --- | --- | --- | --- | --- | --- | --- |
| Middle elevation (m) | 1300 | 1850 | 2250 | 2650 | 2700 | 3050 | 3400 | 3900 | 4350 |
| Elevational range (m) | 600 | 500 | 300 | 500 | 400 | 300 | 400 | 600 | 300 |
| Latitude (N) (degree) | 29.42 ~ 29.57 | | | | 26.87 ~ 28.87 | | | | |
| Longitude (E) (degree) | 102.82 ~ 103 | | | | 99.33 ~ 100.48 | | | | |
| Reference | *c.* | | | | *l.* | | | | |
| *Chiropodomys gliroides* | - | - | - | - | - | - | - | - | - |
| *Apodemus chevrieri* | √ | √ | √ | √ | √ | √ | √ | - | - |
| *Apodemus peninsulae* | - | - | - | - | - | - | - | - | - |
| *Apodemus draco* | √ | √ | √ | √ | √ | √ | √ | √ | √ |
| *Apodemus latronum* | - | - | - | - | - | √ | √ | √ | √ |
| *Mus caroli* | - | - | - | - | - | - | - | - | - |
| *Mus musculus* | - | - | - | - | - | - | - | - | - |
| *Mus pahari* | - | - | - | - | - | - | - | - | - |
| *Bandicota indica* | - | - | - | - | - | - | - | - | - |
| *Rattus losea* | - | - | - | - | - | - | - | - | - |
| *Rattus rattus* | - | - | - | - | - | - | - | - | - |
| *Rattus tanezumi* | - | - | - | - | - | - | - | - | - |
| *Rattus nitidus* | - | - | - | - | √ | √ | √ | - | - |
| *Rattus norvegicus* | √ | - | - | - | - | - | - | - | - |
| *Berylmys bowersi* | - | - | - | - | - | - | - | - | - |
| *Niviventer andersoni* | √ | √ | √ | - | - | - | √ | √ | - |
| *Niviventer excelsior* | √ | √ | - | - | - | - | - | - | - |
| *Niviventer confucianus* | √ | √ | √ | - | √ | √ | √ | √ | - |
| *Niviventer eha* | - | - | - | - | - | - | - | - | - |
| *Niviventer fulvescens* | - | - | - | - | - | - | - | - | - |
| *Niviventer ling* | - | - | - | - | - | - | - | - | - |
| *Niviveneter brahma* | - | - | - | - | - | - | - | - | - |
| *Leopoldamys edwardsi* | - | - | - | - | - | - | - | - | - |
| *Micromys minutus* | - | - | - | - | - | - | - | - | - |
| *Caryomys eva* | - | - | - | - | - | - | - | - | - |
| *Eothenomys miletus* | - | - | - | - | √ | - | - | - | - |
| *Eothenomys melanogaster* | - | √ | √ | √ | - | - | - | - | - |
| *Eothenomys chinensis* | - | √ | √ | √ | - | - | - | - | - |
| *Eothenomys custos* | - | - | - | - | - | √ | √ | √ | - |
| *Eothenomyswardi* | - | - | - | - | - | - | - | - | - |
| *Eothenomys olitor* | - | - | - | - | - | - | - | - | - |
| *Eothenomys proditor* | - | - | - | - | - | - | - | - | - |
| *Microtus limnophilus* | - | √ | √ | - | - | - | - | - | - |
| *Microtus clarkei* | - | - | - | - | - | - | - | √ | √ |
| *Neodon irene* | - | √ | √ | √ | - | - | - | - | - |
| *Microtus oeconomus* | - | - | - | √ | - | - | - | - | - |
| *Volemys musseri* | - | - | - | - | - | - | - | - | - |
| *Dremomys lokriah* | - | - | - | - | - | - | - | - | - |
| *Dremomys pernyi* | - | - | - | - | - | - | - | √ | - |
| *Tamiops swinhoei* | - | - | - | - | - | - | - | √ | √ |
| *Sciurotamias davidianus* | - | - | - | - | - | - | - | - | - |
| *Petaurista xanthotis* | - | - | - | - | - | - | - | √ | - |
| *Rhizomys sinensis* | - | - | - | - | - | - | - | - | - |
| *Eozapus setchuanus* | - | - | - | - | - | √ | √ | √ | - |
| *Sicista concolor* | - | - | - | - | - | - | - | - | - |

Continued

|  | Xianggujing (XGJ) | | | | | | Yulong Mountian (YLM) | | | |
| --- | --- | --- | --- | --- | --- | --- | --- | --- | --- | --- |
| Middle elevation (m) | 2500 | 2800 | 3100 | 3400 | 3700 | 3950 | 1900 | 3000 | 3450 | 4150 |
| Elevational range (m) | 100 | 100 | 100 | 100 | 100 | 100 | 200 | 200 | 700 | 600 |
| Latitude (N) (degree) | 27.62 | 27.64 | 27.64 | 27.65 | 27.66 | 27.68 | 27.17 ~ 27.67 | | | |
| Longitude (E) (degree) | 99.37 | 99.37 | 99.37 | 99.37 | 99.37 | 99.38 | 100.17 ~ 100.33 | | | |
| Reference | *field survey (f.)* | | | | | | *b.* | | | |
| *Chiropodomys gliroides* | - | - | - | - | - | - | - | - | - | - |
| *Apodemus chevrieri* | √ | √ | √ | √ | √ | √ | √ | √ | √ | - |
| *Apodemus peninsulae* | √ | √ | √ | √ | √ | √ | - | - | - | - |
| *Apodemus draco* | √ | √ | √ | √ | √ | √ | - | √ | √ | √ |
| *Apodemus latronum* | √ | √ | √ | √ | √ | √ | - | √ | √ | √ |
| *Mus caroli* | - | - | - | - | - | - | - | - | - | - |
| *Mus musculus* | - | - | - | - | - | - | √ | - | - | - |
| *Mus pahari* | - | - | - | - | - | - | - | - | - | - |
| *Bandicota indica* | - | - | - | - | - | - | - | - | - | - |
| *Rattus losea* | - | - | - | - | - | - | - | - | - | - |
| *Rattus rattus* | - | - | - | - | - | - | √ | - | - | - |
| *Rattus tanezumi* | - | √ | - | - | - | - | - | - | - | - |
| *Rattus nitidus* | - | - | - | - | - | - | √ | √ | √ | - |
| *Rattus norvegicus* | - | - | - | - | - | - | √ | √ | √ | - |
| *Berylmys bowersi* | - | - | - | - | - | - | - | - | - | - |
| *Niviventer andersoni* | - | √ | √ | √ | - | - | - | √ | √ | √ |
| *Niviventer excelsior* | - | - | - | - | - | - | - | - | - | - |
| *Niviventer confucianus* | √ | √ | √ | - | - | - | - | √ | √ | - |
| *Niviventer eha* | - | - | - | - | - | √ | - | - | - | - |
| *Niviventer fulvescens* | - | - | - | - | - | - | - | - | - | - |
| *Niviventer ling* | - | - | - | - | - | - | - | - | - | - |
| *Niviveneter brahma* | - | - | - | - | - | - | - | - | - | - |
| *Leopoldamys edwardsi* | - | - | - | - | - | - | - | - | - | - |
| *Micromys minutus* | √ | - | - | - | - | - | √ | - | - | - |
| *Caryomys eva* | - | - | - | - | - | - | - | - | - | - |
| *Eothenomys miletus* | √ | - | - | - | - | - | - | √ | - | - |
| *Eothenomys melanogaster* | - | - | - | - | - | - | - | - | - | - |
| *Eothenomys chinensis* | - | - | - | - | - | - | - | - | - | - |
| *Eothenomys custos* | √ | √ | √ | √ | √ | √ | - | √ | √ | √ |
| *Eothenomyswardi* | - | - | - | - | - | - | - | - | - | - |
| *Eothenomys olitor* | - | - | - | - | - | - | - | - | - | - |
| *Eothenomys proditor* | - | - | - | - | - | - | - | √ | √ | - |
| *Microtus limnophilus* | - | - | - | - | - | - | - | - | - | - |
| *Microtus clarkei* | - | - | - | - | - | - | - | - | - | - |
| *Neodon irene* | - | - | - | - | √ | √ | - | - | - | - |
| *Microtus oeconomus* | - | - | - | - | - | - | - | - | - | - |
| *Volemys musseri* | - | - | - | - | - | - | - | - | - | - |
| *Dremomys lokriah* | - | - | - | - | - | - | - | - | - | - |
| *Dremomys pernyi* | - | - | - | - | - | - | - | - | - | - |
| *Tamiops swinhoei* | √ | √ | √ | - | - | - | - | - | - | - |
| *Sciurotamias davidianus* | √ | √ | √ | - | - | - | - | - | - | - |
| *Petaurista xanthotis* | - | - | - | - | - | - | - | - | - | - |
| *Rhizomys sinensis* | - | - | - | - | - | - | - | - | - | - |
| *Eozapus setchuanus* | - | - | - | - | - | - | - | - | - | - |
| *Sicista concolor* | - | - | - | - | - | - | - | - | - | - |

Continued

|  | Laojun Mountian-Yunnan (LJM) | | | Eastern Gaoligong Mountian (EGL) | | | | Western Gaoligong Mountain (WGL) | | | |
| --- | --- | --- | --- | --- | --- | --- | --- | --- | --- | --- | --- |
| Middle elevation (m) | 3250 | 3750 | 4123 | 2250 | 2750 | 3250 | 3800 | 2075 | 2700 | 3000 | 3300 |
| Elevational range (m) | 500 | 500 | 247 | 500 | 500 | 600 | 400 | 750 | 500 | 150 | 400 |
| Latitude (N) (degree) | 26.50 ~ 26.87 | | | 24.93 ~ 28.37 | | | | 26.87 ~ 28.87 | | | |
| Longitude (E) (degree) | 99.70 ~ 100.43 | | | 98.60 ~ 99.33 | | | | 99.33 ~ 99.48 | | | |
| Reference | *h.* | | | *k.* | | | | *a.* | | | |
| *Chiropodomys gliroides* | - | - | - | - | - | - | - | - | - | - | - |
| *Apodemus chevrieri* | √ | √ | √ | - | - | - | - | - | - | - | - |
| *Apodemus peninsulae* | √ | √ | - | - | - | - | - | - | - | - | - |
| *Apodemus draco* | √ | √ | - | - | - | - | - | √ | √ | √ | √ |
| *Apodemus latronum* | - | - | - | √ | √ | √ | √ | - | - | - | - |
| *Mus caroli* | - | - | - | - | - | - | - | - | - | - | - |
| *Mus musculus* | - | - | - | - | - | - | - | - | - | - | - |
| *Mus pahari* | - | - | - | - | - | - | - | √ | - | - | - |
| *Bandicota indica* | - | - | - | - | - | - | - | - | - | - | - |
| *Rattus losea* | - | - | - | - | - | - | - | - | - | - | - |
| *Rattus rattus* | - | - | - | - | - | - | - | - | - | - | - |
| *Rattus tanezumi* | - | - | - | - | - | - | - | - | - | - | - |
| *Rattus nitidus* | √ | - | - | - | - | - | - | √ | √ | - | - |
| *Rattus norvegicus* | √ | - | - | - | - | - | - | - | - | - | - |
| *Berylmys bowersi* | - | - | - | - | - | - | - | - | - | - | - |
| *Niviventer andersoni* | √ | √ | - | √ | √ | - | - | - | - | - | - |
| *Niviventer excelsior* | - | - | - | - | - | - | - | - | √ | √ | √ |
| *Niviventer confucianus* | √ | √ | - | √ | √ | √ | - | - | √ | √ | - |
| *Niviventer eha* | - | - | - | - | √ | √ | - | - | - | √ | √ |
| *Niviventer fulvescens* | - | - | - | √ | - | - | - | - | - | - | - |
| *Niviventer ling* | - | - | - | - | - | - | - | - | - | - | - |
| *Niviveneter brahma* | - | - | - | - | - | - | - | - | √ | √ | - |
| *Leopoldamys edwardsi* | - | - | - | - | - | - | - | - | - | - | - |
| *Micromys minutus* | - | - | - | - | - | - | - | - | - | - | - |
| *Caryomys eva* | - | - | - | - | - | - | - | - | - | - | - |
| *Eothenomys miletus* | √ | √ | - | - | - | - | - | - | - | - | - |
| *Eothenomys melanogaster* | - | - | - | √ | √ | √ | - | - | √ | - | - |
| *Eothenomys chinensis* | - | - | - | - | - | - | - | - | - | - | - |
| *Eothenomys custos* | √ | √ | √ | - | - | - | - | - | - | - | - |
| *Eothenomyswardi* | - | - | - | - | - | - | - | - | √ | √ | √ |
| *Eothenomys olitor* | - | - | - | - | - | - | - | - | - | - | - |
| *Eothenomys proditor* | - | - | - | - | - | - | - | - | - | - | - |
| *Microtus limnophilus* | - | - | - | - | - | - | - | - | - | - | - |
| *Microtus clarkei* | - | - | - | - | - | √ | √ | - | - | - | - |
| *Neodon irene* | - | - | - | - | - | √ | √ | - | - | - | - |
| *Microtus oeconomus* | - | - | - | - | - | - | - | - | - | - | - |
| *Volemys musseri* | - | - | - | - | - | - | - | - | - | - | - |
| *Dremomys lokriah* | - | - | - | - | √ | - | - | - | - | - | - |
| *Dremomys pernyi* | - | - | - | - | - | - | - | - | - | - | - |
| *Tamiops swinhoei* | - | - | - | √ | - | - | - | - | - | - | - |
| *Sciurotamias davidianus* | - | - | - | - | - | - | - | - | - | - | - |
| *Petaurista xanthotis* | - | - | - | - | - | - | - | - | - | - | - |
| *Rhizomys sinensis* | - | - | - | - | - | - | - | - | - | - | - |
| *Eozapus setchuanus* | - | - | - | - | - | - | - | - | - | - | - |
| *Sicista concolor* | - | - | - | - | - | - | - | - | - | - | - |

Continued

|  | Wuliang Mountain (WLM) | | | | Baicaoling (BCL) | | |
| --- | --- | --- | --- | --- | --- | --- | --- |
| Middle elevation (m) | 1600 | 2350 | 2815 | 3118 | 2900 | 3375 | 3587 |
| Elevational range (m) | 800 | 700 | 230 | 376 | 500 | 350 | 157 |
| Latitude (N) (degree) | 24.00 ~ 24.75 | | | | 26.15 | | |
| Longitude (E) (degree) | 100.42 ~ 100.88 | | | | 101.22 | | |
| Reference | *j.* | | | | *i.* | | |
| *Chiropodomys gliroides* | √ | √ | √ | √ | - | - | - |
| *Apodemus chevrieri* | - | - | - | - | √ | √ | √ |
| *Apodemus peninsulae* | - | - | - | - | - | - | - |
| *Apodemus draco* | - | √ | √ | √ | √ | √ | √ |
| *Apodemus latronum* | - | - | - | - | √ | √ | √ |
| *Mus caroli* | √ | - | - | - | - | - | - |
| *Mus musculus* | - | - | - | - | - | - | - |
| *Mus pahari* | √ | √ | - | - | - | - | - |
| *Bandicota indica* | √ | - | - | - | - | - | - |
| *Rattus losea* | - | - | - | - | - | - | - |
| *Rattus rattus* | - | - | - | - | - | - | - |
| *Rattus tanezumi* | √ | √ | - | - | - | - | - |
| *Rattus nitidus* | - | - | - | - | - | - | - |
| *Rattus norvegicus* | - | - | - | - | √ | - | - |
| *Berylmys bowersi* | √ | - | - | - | - | - | - |
| *Niviventer andersoni* | - | - | √ | √ | √ | √ | √ |
| *Niviventer excelsior* | - | - | - | - | - | - | - |
| *Niviventer confucianus* | √ | √ | - | - | √ | √ | √ |
| *Niviventer eha* | - | - | √ | √ | - | - | - |
| *Niviventer fulvescens* | √ | √ | - | - | - | - | - |
| *Niviventer ling* | - | - | - | - | - | - | - |
| *Niviveneter brahma* | - | - | - | - | - | - | - |
| *Leopoldamys edwardsi* | - | - | - | - | - | - | - |
| *Micromys minutus* | - | - | - | - | √ | √ | √ |
| *Caryomys eva* | - | - | - | - | - | - | - |
| *Eothenomys miletus* | - | √ | √ | √ | √ | √ | √ |
| *Eothenomys melanogaster* | - | - | - | - | - | - | - |
| *Eothenomys chinensis* | - | - | - | - | - | - | - |
| *Eothenomys custos* | - | - | - | - | - | - | - |
| *Eothenomyswardi* | - | - | - | - | - | - | - |
| *Eothenomys olitor* | - | - | - | - | √ | √ | √ |
| *Eothenomys proditor* | - | - | - | - | - | - | - |
| *Microtus limnophilus* | - | - | - | - | - | - | - |
| *Microtus clarkei* | - | - | - | - | - | - | - |
| *Neodon irene* | - | - | - | - | - | - | - |
| *Microtus oeconomus* | - | - | - | - | - | - | - |
| *Volemys musseri* | - | - | - | - | - | - | - |
| *Dremomys lokriah* | - | - | - | - | - | - | - |
| *Dremomys pernyi* | - | - | - | - | - | - | - |
| *Tamiops swinhoei* | - | - | - | - | - | √ | - |
| *Sciurotamias davidianus* | - | - | - | - | - | - | - |
| *Petaurista xanthotis* | - | - | - | - | - | - | - |
| *Rhizomys sinensis* | - | - | - | - | - | - | - |
| *Eozapus setchuanus* | - | - | - | - | - | - | - |
| *Sicista concolor* | - | - | - | - | - | - | - |

Reference in **Table S1**:

*a*). Gong, Z.-d. et al. 2001a. The vertical distribution and community structure of small mammal hosts of RSSE nature foci in Mt. Gaoligong, Yunnan. Endemic Diseases Bulletin 16: 23-26. (In chinese)

*b*). Gong, Z.-d., H.-y. Wu, and X.-d. Duan. 2001b. Clustering analysis and fauna study on small mammal fauna in Yulong Mt. Nature Reserve, Yunnan. Endemic Diseases Bulletin 16: 67-73. (In chinese)

*c*). Qi, D. et al. 2005. Structure of community of small mammals in Wawushan Natural Reserve, Sichuan Province. China, J. China West Normal Univ. (Nat. Sci.) 26: 14-18. (In chinese)

*d*). Sun, Z. et al. 2013. The faunal composition and distribution of small mammals in Erlang Mountains. Acta Theriologica Sinica 33: 82-89. (In chinese)

*e*). Tu, F. et al. 2012. Fauna and species diversity of small mammals in Jiajin Mountains, Sichuan Province, China. Acta Theriologica Sinica 32: 287-296.

*f*). Wen, Z. et al. 2014. Seasonal Change of Species Diversity Patterns of Non‐volant Small Mammals along Three Subtropical Elevational Gradients. Biotropica 46: 479-488.

*g*). Wu, Y. et al. 2013. What drives the species richness patterns of non‐volant small mammals along a subtropical elevational gradient? Ecography 36: 185-196.

*h*). Yang, G., and K. Tao. 1986. The vertical distribution of rodents of Laojun Mountain, Yunnan. Zoological Research 7: 311-316. (In chinese)

*i*). Zhang, Y. et al. 2001. The Community Structure and Vertical Distribution of Small Mammal in Baicaoling Mt., Yunnan Province, China. Chinese Journal of Zoology 37: 63-66. (In chinese)

*j*). Zhang, Y., Z. Gong, and H. Wu. 2005. Community structure and vertical distribution of small mammals in Wuliang Mountain Nature Reserve, Yunnan province, China. ENDEMIC DISEASES BULLETIN 20: 13. (In chinese)

*k*). Gong Zhengda, and Xie Baoqi. 1989. Field survey on small mammals of Gaoli Mountain. Chinese Journal of Zoology 24: 28-32. (In chinese)

*l*). Liu et al. 2013. Research on the vertical distribution of small mammals in Shangri-la. Chinese Journal of Zoology: 619-625. (In chinese)
