# Supplementary material for: The roles of environment, space, and phylogeny in determining functional dispersion of rodents (Rodentia) in the Hengduan Mountains, China
Source: Ecol Evol. 2017 Nov 12;7(24):10941–51. doi: 10.1002/ece3.3613 (PMC5743695; doi:10.1002/ece3.3613)
Supplement: Supplementary file 2 [file ECE3-7-10941-s002.docx]

**Table S2.** DNA sequence and accession numbers in Genebank database.

| **Species** | **Accession numbers for DNA sequences** | | | | | | |
| --- | --- | --- | --- | --- | --- | --- | --- |
|  | **Cytb** | **12s-RNA** | **16s-RNA** | **CO1** | **IRBP** | **RAG1** | **GHR** |
| *Chiropodomys gliroides* | EU349740 |  |  | JF444993 | KJ607299 | EU349882 | KJ607289 |
| *Apodemus chevrieri* | AB096821 |  |  | KF999113 |  |  |  |
| *Apodemus peninsulae* | AB073811 | HQ660074 | HQ660074 | KC861411 |  |  |  |
| *Apodemus draco* | AB109397 | NC_019584 | NC_019584 | HQ020517 | JQ043403 |  |  |
| *Apodemus latronum* | AB096834 | HQ333256 | HQ333256 | HQ318705 |  |  | GU908448 |
| *Mus caroli* | AB253438 | AY057798 |  |  |  | AB125822 |  |
| *Mus musculus* | AB819920 | LC062084 | LC062084 |  | JX457617 | AB125834 |  |
| *Mus pahari* | AB096839 | AB125793 |  | JF445031 |  |  | KC953280 |
| *Bandicota indica* | JQ735467 |  | JQ843689 | FR775844 | HM217713 |  |  |
| *Rattus losea* | HM031721 |  |  | JF445255 |  |  |  |
| *Rattus rattus* | AB211039 | AJ005780 | JQ844107 | JX426131 | HM217754 |  | AM910976 |
| *Rattus tanezumi* | AB211043 | JQ287758 |  | KF999137 | JX534162 | KM397346 | KM397260 |
| *Rattus nitidus* | FR775884 |  |  | KR996517 | HM217716 |  |  |
| *Rattus norvegicus* | AB746366 | AB183258 |  |  | AJ429134 | AB125848 | JF412704 |
| *Berylmys bowersi* | JX573336 |  |  | KC010296 | KC878201 |  | AM910946 |
| *Niviventer andersoni* | KF740296 |  |  | KF739986 | KF769390 |  |  |
| *Niviventer excelsior* | GU479935 |  |  | KF999099 | KC953419 |  | GQ405386 |
| *Niviventer confucianus* | KF740301 |  |  | KF739943 | KF769394 |  |  |
| *Niviventer eha* | KF740082 |  |  | KF739990 | KF769332 |  |  |
| *Niviventer fulvescens* | KF740278 |  |  | KF739980 | KF769374 |  | JN009859 |
| *Niviventer ling* | KF740038 |  |  | KF739963 |  |  |  |
| *Niviveneter brahma* | EF053011 |  |  |  |  |  |  |
| *Leopoldamys edwardsi* | JQ755928 | NC_025670 | NC_025670 | JQ755854 | JQ755959 |  |  |
| *Micromys minutus* | AB033697 | AJ311139 | NC_027932 | NC_027932 | JX457657 | EU349904 | EU349818 |
| *Caryomys eva* | HM165401 |  |  | HM165280 |  |  |  |
| *Eothenomys miletus* | HM165379 |  |  | HM165357 |  |  |  |
| *Eothenomys melanogaster* | HM165423 |  |  | KF999116 |  |  |  |
| *Eothenomys chinensis* | HM165437 | NC_013571 | NC_013571 | KF999162 |  |  | GQ374497 |
| *Eothenomys custos* | HM165414 |  |  | HM165352 |  |  |  |
| *Eothenomys wardi* | JQ818228 |  |  | JQ818227 |  |  |  |
| *Eothenomys olitor* | AY426690 |  |  |  |  |  |  |
| *Eothenomys proditor* | HM165441 |  |  | HM165288 |  |  |  |
| *Microtus limnophilus* | FJ986323 |  |  | JQ043501 |  |  |  |
| *Microtus clarkei* | AY641526 |  |  |  |  |  |  |
| *Neodon irene* | JF906127 | NC_016055 | NC_016055 | KC709680 | JF906136 | AY241464 | GQ374493 |
| *Microtus oeconomus* | AY219981 | AJ616853 |  |  |  |  | GQ374499 |
| *Volemys musseri* | JF906121 |  |  |  | JF906128 |  | GQ374483 |
| *Dremomys lokriah* | EF539335 |  |  |  |  |  |  |
| *Dremomys pernyi* | HQ698363 |  |  |  | HQ698527 | AY241482 |  |
| *Tamiops swinhoei* | HQ698409 | NC_026875 | NC_026875 | NC_026875 | HQ698548 | HQ698465 |  |
| *Sciurotamias davidianus* | KC005710 | AY227554 | AY227503 |  | AY227621 | AY241488 |  |
| *Petaurista xanthotis* | DQ072111 |  |  |  |  |  |  |
| *Rhizomys sinensis* | AF326274 | KM434232 | KM434232 | KM434232 |  |  |  |
| *Eozapus setchuanus* | KM397177 | NC_027578 | NC_027578 | JX962294 | EU839445 | KM397310 | KM397224 |
| *Sicista concolor* | KM397207 | KJ648496 | KJ648496 | JQ043546 | KM397167 | KM397339 | KM397252 |
| *Ochotona princeps* | AF272989 | AF390540 | AF390540 | NC_005358 | AY057832 | JQ073183 | JF938867 |
