# Supplementary material for: The roles of environment, space, and phylogeny in determining functional dispersion of rodents (Rodentia) in the Hengduan Mountains, China
Source: Ecol Evol. 2017 Nov 12;7(24):10941–51. doi: 10.1002/ece3.3613 (PMC5743695; doi:10.1002/ece3.3613)
Supplement: Supplementary file 3 [file ECE3-7-10941-s003.docx]

**Table S3.** Measurements for functional attributes of 45 rodent species. The data signed with "#" are extracted from history records of Smith *et al.* 2010. Information of animal components in diet is extracted from Kissling *et al*. 2014.

|  | **Body size (mm)** | **Tail/body ratio** | **Animal components in diet ## (0, 1)** |
| --- | --- | --- | --- |
| *Chiropodomys gliroides* | 92 | 1.3 | 0 |
| *Apodemus chevrieri* | 106 | 0.75 | 1 |
| *Apodemus peninsulae* | 101 | 0.95 | 1 |
| *Apodemus draco* | 88 | 1.05 | 1 |
| *Apodemus latronum* | 86.25 | 1.23 | 1 |
| *Mus caroli* | 83.5 | 1 | 1 |
| *Mus musculus* | 71.25# | 0.96# | 1 |
| *Mus pahari* | 95.5 | 0.93 | 1 |
| *Bandicota indica* | 254.75# | 0.91# | 1 |
| *Rattus losea* | 114.75 | 1.05 | 1 |
| *Rattus rattus* | 162.5 | 1.18 | 1 |
| *Rattus tanezumi* | 151.25 | 1.11 | 1 |
| *Rattus nitidus* | 176 | 0.93 | 1 |
| *Rattus norvegicus* | 164.75 | 1.02 | 1 |
| *Berylmys bowersi* | 260.5 | 1.04 | 1 |
| *Niviventer andersoni* | 183 | 1.29 | 1 |
| *Niviventer excelsior* | 119.75 | 1.46 | 1 |
| *Niviventer confucianus* | 139 | 1.27 | 1 |
| *Niviventer eha* | 115 | 1.35 | 1 |
| *Niviventer fulvescens* | 141 | 1.29 | 1 |
| *Niviventer ling* | 139.75 | 1.23 | 1 |
| *Niviveneter brahma* | 153.50# | 1.44# | 1 |
| *Leopoldamys edwardsi* | 274.5 | 1.09 | 1 |
| *Micromys minutus* | 61.75 | 1.11 | 1 |
| *Caryomys eva* | 91 | 0.55 | 1 |
| *Eothenomys miletus* | 95 | 0.42 | 1 |
| *Eothenomys melanogaster* | 101.5 | 0.42 | 1 |
| *Eothenomys chinensis* | 113 | 0.62 | 1 |
| *Eothenomys custos* | 93 | 0.51 | 1 |
| *Eothenomys wardi* | 108.5 | 0.58 | 1 |
| *Eothenomys olitor* | 86 | 0.52 | 1 |
| *Eothenomys proditor* | 110 | 0.44 | 1 |
| *Microtus limnophilus* | 103 | 0.39 | 1 |
| *Microtus clarkei* | 117.00# | 0.53# | 0 |
| *Neodon irene* | 90 | 0.23 | 0 |
| *Microtus oeconomus* | 107 | 1.51 | 0 |
| *Volemys musseri* | 109.50# | 1.63# | 1 |
| *Dremomys lokriah* | 200.00# | 0.84# | 1 |
| *Dremomys pernyi* | 185.75 | 0.79 | 1 |
| *Tamiops swinhoei* | 130 | 0.73 | 1 |
| *Sciurotamias davidianus* | 276.25 | 0.49 | 0 |
| *Petaurista xanthotis* | 336 | 1 | 0 |
| *Rhizomys sinensis* | 327.38 | 0.39 | 1 |
| *Eozapus setchuanus* | 72.75 | 0.27 | 0 |
| *Sicista concolor* | 60.75 | 0.37 | 0 |

Reference in Table S3:

# Smith, A.T., Xie, Y., Hoffmann, R.S., Lunde, D., MacKinnon, J., Wilson, D.E., Wozencraft, W.C. & Gemma, F. (2010) A guide to the mammals of China. Princeton University Press.

## Kissling, W.D., Dalby, L., Flojgaard, C., Lenoir, J., Sandel, B., Sandom, C., Trojelsgaard, K. & Svenning, J.C. (2014) Establishing macroecological trait datasets: digitalization, extrapolation, and validation of diet preferences in terrestrial mammals worldwide. Ecol Evol, 4, 2913-2930.
